# The Median Nerve at the Carpal Tunnel … and Elsewhere

**DOI:** 10.5334/jbsr.1354

**Published:** 2018-01-31

**Authors:** Philippe Meyer, Pierre-Francois Lintingre, Lionel Pesquer, Nicolas Poussange, Alain Silvestre, Benjamin Dallaudière

**Affiliations:** 1 MSK Imaging Department, Clinique du Sport de Bordeaux-Mérignac, FR

**Keywords:** nerve, median, neuropathy, US, MRI

## Abstract

The median nerve (MN) may be affected by various peripheral neuropathies, each of which may be categorized according to its cause, as either an extrinsic (due to an entrapment or a nerve compression) or an intrinsic (including neurogenic tumors) neuropathy. Entrapment neuropathies are characterized by alterations of the nerve function that are caused by mechanical or dynamic compression. It occurs because of anatomic constraints at specific locations including sites where the nerve courses through fibro-osseous or fibromuscular tunnels or penetrates a muscle. For the diagnosis of peripheral neuropathies, physicians traditionally relied primarily on clinical findings and electrodiagnostic testing with electromyography. However, if further doubt exists, clinicians may ask for an additional imaging evaluation.

## 1. Anatomy

The median nerve arises from the anteromedial and anterolateral cords of the brachial plexus and is innervated by the C6, C7, C8 and T1 nerve roots. After originating from the brachial plexus in the axilla, the MN lies laterally close to the brachial artery and then crosses it anteriorly to medially by drawing a “S-Shape”. After entering the cubital fossa, the MN passes beneath the bicipital aponeurosis (also known as lacertus fibrosus), over the brachialis muscle and then between the two heads of the pronator teres.

As the nerve enters the anterior antebrachial compartment, it runs under the aponeurotic arch of the flexor digitorum superficialis before coursing between the flexor digitorum superficialis and profundus muscles.

The MN passes under the flexor retinaculum and into the carpal tunnel running anteriorly and laterally to the tendons of the flexor digitorum superficialis. Distal to the carpal tunnel, it gives off a motor branch (for the thenar compartment and the first and second lumbricals) and a sensory branch which subdivides into four digital palmar branches.

The median nerve only provides motor function to the forearm, and motor and sensory function to the wrist and hand.

The median nerve is also responsible for the cutaneous innervation of part of the hand including the thenar eminence, the lateral side of the palm, the palmar side of the index, the thumb and middle finger and the dorsal side of the distal phalanx of index and middle finger and half the ring finger.

Median nerve mononeuropathy is the most common peripheral nerve neuropathy. Although the anatomic course of the MN is largely protected, a compression of the nerve can occur in particular at the joints. The carpal tunnel is the most common site of compression. However in 7 to 10% of cases entrapment of the median nerve occurs at various sites along its course [1]. Actually, from proximal to distal, the median nerve can also be entrapped at four locations around the elbow, including:

– The supracondylar process continued by the ligament of Struthers,
– The lacertus fibrosus (where the course of the MN is superficial),
– Between the humeral and the ulnar heads of the pronator teres muscle,
– The fibrous arch of the origin of the flexor digitorum superficialis.

In the proximal part of the forearm, the anterior interosseous nerve, which is the largest branch of the median nerve, can be compressed.

## 2. Supracondylar Process Syndrome

The supracondylar process syndrome is a very rare neuropathy that affects the median nerve (0.5% of cases) [2].

**Figure 1:**
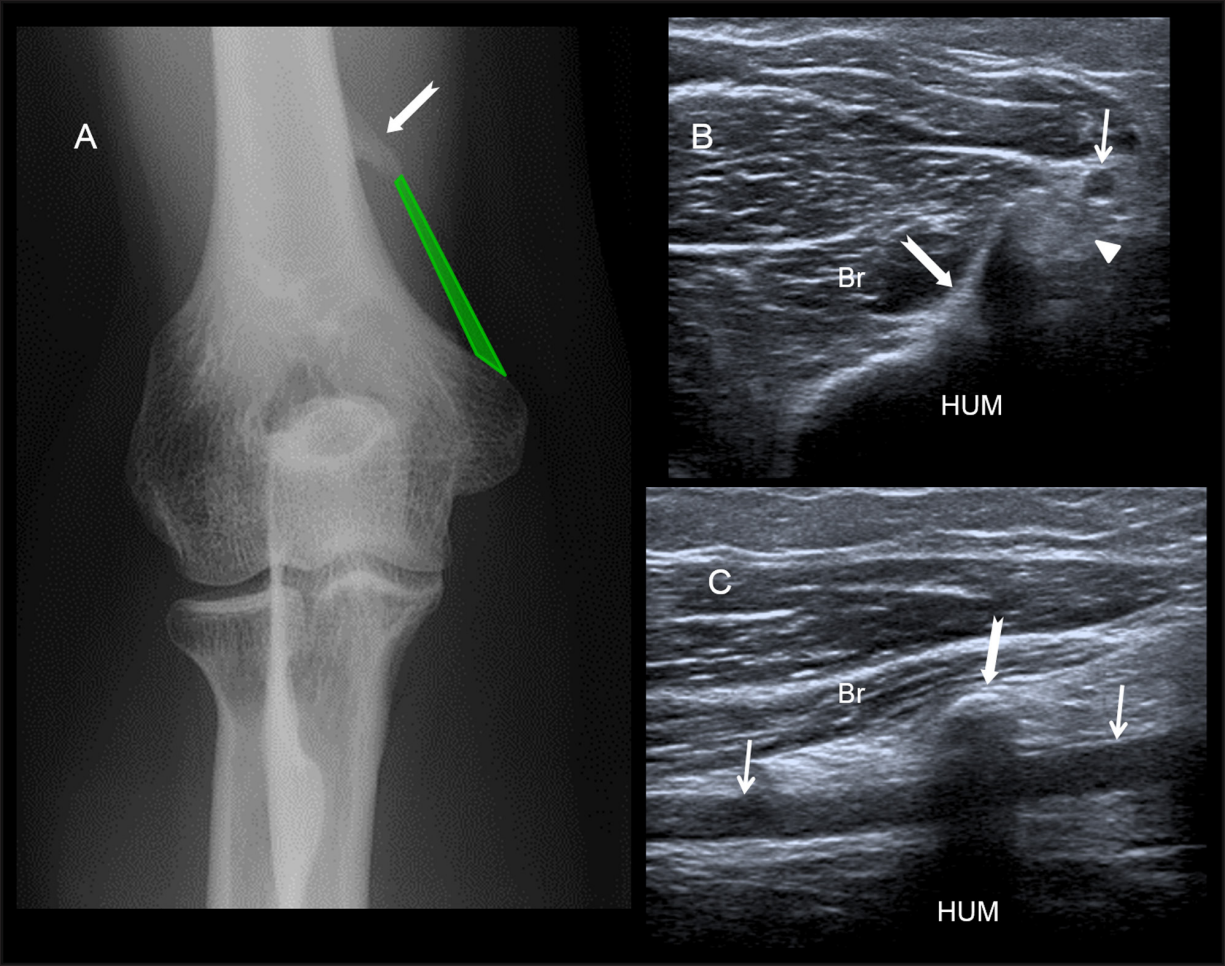
Supracondylar process on US examination. **A)** Posteroanterior radiographic view and US examination both on axial **B)** and longitudinal **C)** views. Br = Brachialis muscle, HUM = Humeral diaphysis, B = Biceps muscle. The bony spur is located anteromedially at the distal humerus. The ligament of Struthers (in green) connects the supracondylar process (white arrow) to the medial epicondyle, encasing neurovascular structures including the brachial artery (small white arrows) and the median nerve (white arrowhead) which may be entrapped.

The supracondylar process is a congenital bony spur located medially at the distal humerus, approximately 4–8 cm proximal to the medial epicondyle and 2–20 mm long. The ligament of Struthers is a fibrous band connecting the supracondylar process to the medial epicondyle, encasing neurovascular structures [3] including the median nerve and the brachial artery but also the ulnar nerve, the ulnar artery and a branch of the musculocutaneous nerve. This congenital osteofibrous arcade can cause a median nerve neuropathy which can be acute in case of fracture [2,4]. Anatomical variations are described regarding the course of the nerve which can pass beneath or over the ligament of Struthers. The pronator teres and more rarely coracobrachialis muscles may insert at the supracondylar process. Apart from the supracondylar process, MR and US images may show an anatomic relationship with the median nerve (Figure 1).

Dynamic examination (in particular extension of the elbow and pronation) may provoke symptoms such as increased pain. Typically, in the vast majority of cases the symptoms regress with rest and anti-inflammatory analgesics. In the rare resistant cases, a surgical treatment is indicated consisting of the removal of both the supracondylar process and the ligament of Struthers [2].

## 3. Lacertus Fibrosus

**Figure 2:**
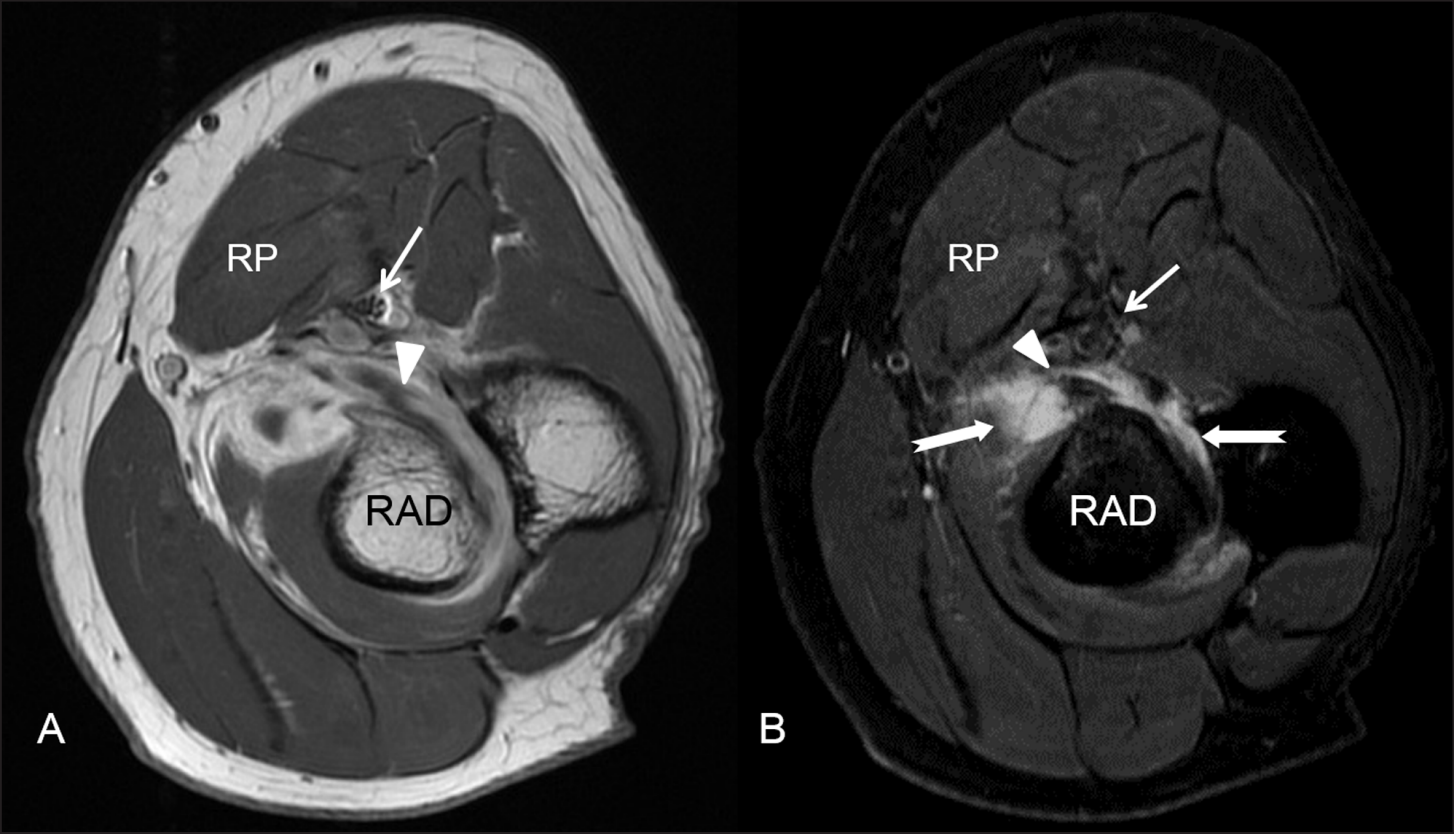
Median nerve entrapment neuropathy caused by an extrinsic compression due to a bicipital bursa. MRI with axial views: T1-weighted **A)** and T2-Weighted Fat Sat sequences **B)**. RP = pronator teres muscle, RAD = radius. The median nerve (small white arrow) is located close to the bursa (white arrows) surrounding the insertion of the distal biceps tendon on the radial tuberosity.

The superficial bicipital aponeurosis extends distally with the so-called lacertus fibrosus. This aponeurotic prolongation extends from the myotendinous junction of the distal biceps to the medial deep fascia of the forearm close to the epicondylar muscles. The “lacertus fibrosus” covers the median nerve and the brachial artery and its function is to keep the biceps tendon in the appropriate position [5]. By enabling real-time dynamic evaluation, US can be useful for the detection of such a dynamic compression of the median nerve by the lacertus fibrosus [4]. Exceptionally, surgery (i.e. neurolysis) may be considered when disabling symptoms remain despite suitable medical treatment (Figure 2).

## 4. Pronator Syndrome

**Figure 3:**
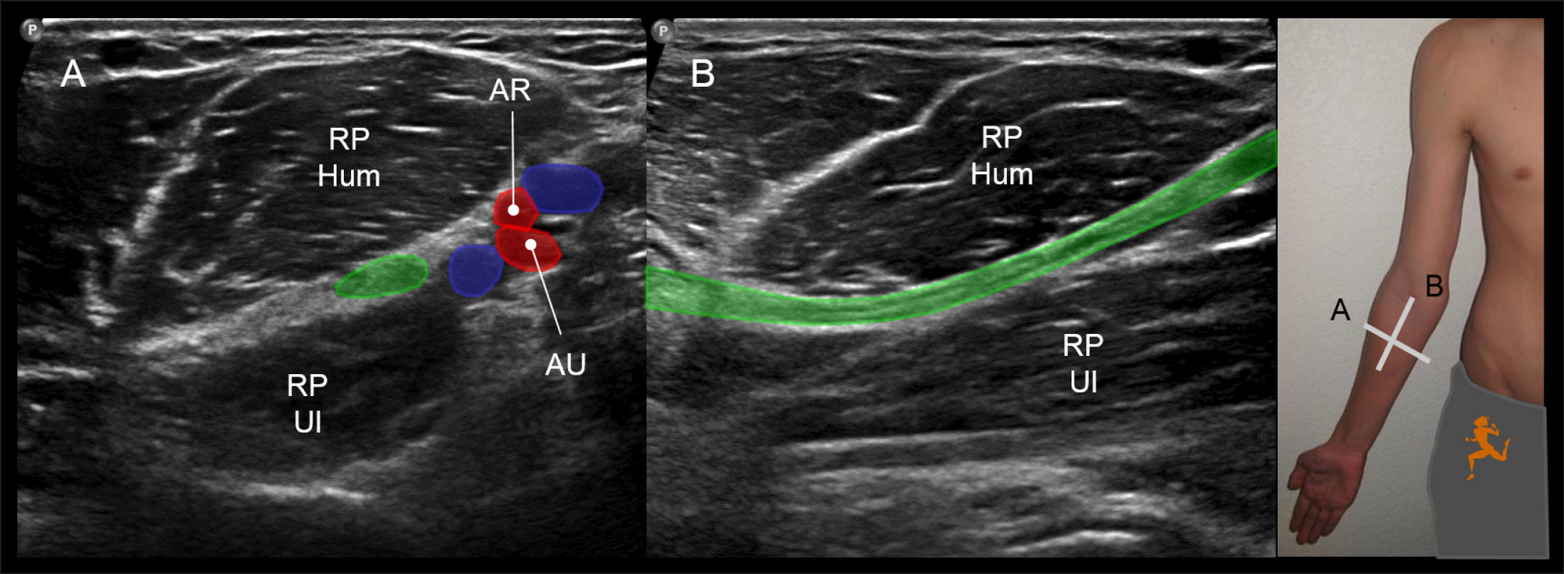
US depicting the median nerve at the level of the pronator teres muscle: axial **A)** and sagittal **B)** views. RP Hum = superficial (or humeral) head of the pronator teres muscle. RP Hum = deep (or ulnar) head of the pronator teres muscle. AR = radial artery, AU = ulnar artery. The median nerve (in green) courses between the humeral (RP Hum) and the ulnar (RP Ul) heads of the pronator teres muscle, beneath the division of the brachial artery into its ulnar (AU) and radial (AR) branches.

Pronator syndrome results from entrapment of the median nerve between the heads of the pronator teres muscle or at the fibrous arch of the origin of the flexor digitorum superficialis passing between its humeral and ulnar heads (formerly called “sublimis bridge” and also known as the arcade of Fearn and Goodfellow [6]) (Figure 3). Patients with pronator syndrome experience non-specific symptoms mimicking carpal tunnel syndrome. However, some clinical findings suggest more specifically a pronator syndrome. Paresthesia of the thenar eminence is evocative of involvement of the palmar cutaneous branch of the median nerve which is typically respected in carpal tunnel syndrome [7,8].

In contrast, a compression at the level of the pronator teres muscle should be considered when repetitive pronation-supination triggers paresthesia. On the other hand, symptom reproduction during resisted contraction of the flexor superficialis is suggestive of an entrapment beneath the arch of the flexor digitorum superficialis [7]. Finally, a few cases of nerve entrapment syndrome are described without any anatomic constraints nor specific location [9].

US examination with the “up and down scanning” technique can reveal an enlargement of the nerve which may be difficult to visualize due to the deep location of the nerve. MR imaging can readily demonstrate early abnormalities such as neurogenic muscle edema. T2-weighted fat-suppressed or STIR image sequences are particularly sensitive in depicting muscle edema involving the pronator teres, the flexor carpi radialis, palmaris longus and the flexor digitorum superficialis muscles [8].

Conservative treatment is effective in 50 to 70% of the patients. In the case of mass lesions and absence of improvement, a surgical decompression is proposed with excellent outcomes (studies show a success rate of 90%) [7].

## 5. Anterior Interossesous Nerve Syndrome

The anterior interosseous nerve is the largest branch of the MN. It originates from the radial side (60% of cases) or from the posterior side (40%) of the MN [10]. The anterior interosseous branch comes from a posterior fascicle of the MN which can be isolated from the brachial plexus.

The clinical presentation can be confounded by numerous normal anatomic variations. In particular, the Martin-Gruber anastomosis is a network of cross connections between motor branches of the anterior interosseous and ulnar nerves, reported in 23% to 40% of the population [3,11].

The anterior interosseous nerve accompanies the anterior interosseous artery along the anterior side of the interosseous membrane of the forearm in the interval between the flexor digitorum profundus and the flexor pollicis longus muscles. Then the nerve ends in the pronator quadratus.

The anterior interosseous nerve syndrome is rare (representing less than 1% of all the entrapment syndromes of the upper limb) [10].

Patients present motor weakness typically manifested by weakened ability to pinch the thumb and index finger together, tested by asking the patient to make an “OK” sign with the hand, reflecting the palsy of the flexor pollicis longus muscle and flexor digitorum profundus muscle to the index finger.

The syndrome may be categorized according to its cause, as either post-traumatic (fractures, surgery, poorly applied casts) or non traumatic. Nerves can be entraped by tendinous or muscular variations (including an accessory head of the flexor pollicis longus also known as Gantzer muscle), bicipital bursa, vascular abnormalities or Volkmann’s ischaemic contractures [10,12].

The observations by magnetic resonance neurography of selective fascicular lesions following motor somatotopy clearly suggest that anterior interosseous nerve syndrome is in fact a multifocal motor fascicular neuropathy of the median nerve trunk [13]. Unfortunately, clinical evaluation is unable to discriminate between the two etiologies.

Gantzer described two different anomalous bellies in the deep flexor region of the forearm, inserting into either the flexor pollicis longus or the flexor digitorum profundus muscles. The incidence of the accessory head of the flexor pollicis longus has been reported to range from 46% to 74% and is more frequent in black people. The flexor digitorum profundus accessory head is less frequently observed (14%). Both accessory muscles are between the median nerve and the anterior interosseous nerve, with the median nerve lying anteriorly and the anterior interosseous posteriorly to the muscle. So a nerve can be entrapped due to repetitive contraction of Gantzer muscle [14,15,16].

Electrodiagnostic studies are often normal but may lately reveal denervation of the affected muscles [17].

The goals of imaging are: 1) To detect an entrapment syndrome, 2) to study the anatomical variations of flexor tendons and 3) to appreciate the intensity of the specific muscle denervation pattern.

An Increased echogenicity and overall reduction in the size of the muscles are key signs consistent with denervation and atrophy induced by neuropathy [18].

MR imaging offers a better depiction of deeper nerves and higher contrast resolution. The field of view must be large enough to include the distal part of the humerus in order to detect supracondylar process. Since the nerve and the injury to it cannot always be visualized even with high-resolution MR imaging, the presence and the pattern of signal changes within muscles may be key in the diagnosis and localization of nerve dysfunction. MR imaging can readily demonstrate abnormalities such as neurogenic muscle edema with T2-weighted or STIR sequences. Neurogenic muscle edema typically affects the entire muscle in a diffuse and homogeneous distribution. In addition only the muscles innervated by the involved nerve are affected. Neurogenic edema occurs in acute and subacute stages, as early as 24 hours after denervation. When axonal lesions persist, muscle atrophy occurs within the first week. Then fatty muscle infiltration evolves irreversibly, from the first month after denervation [3,19,20,21,22]. STIR sequences are particularly sensitive (84%) and specific (100%) in depicting neurogenic muscle edema [21]. In patients with anterior interosseous nerve syndrome, the key MRI finding is a neurogenic edema of the pronator quadratus muscle [3,11]. However an increased non specific signal intensity can be seen idiopathically in this muscle [23].

Conservative management is effective in most of the cases. Surgical decompression is rarely indicated in cases refractory to medical management [24,25,7,10].

## 6. Carpal Tunnel Syndrome

### 6.1. General Points

Carpal tunnel syndrome is the most common peripheral neuropathy of the upper extremities (for example about hundred times more frequent than median nerve compression at the elbow). Prevalence ranges from 2 to 3% in the general population [26] and the incidence may rise as high as 50 cases per 1000 subjects per year [27].

Numerous risk factors are described in literature. Among them, the most common general conditions linked to carpal tunnel syndrome are:

– Diabetes mellitus: the prevalence of clinical carpal tunnel syndrome is 14% in diabetic subjects and 30% in those with diabetic polyneuropathy.
– Pregnancy: symptoms often disappear spontaneously after delivery.
– Obesity and Hypothyroidism.
– Rheumatoid arthritis, Chronic gout, Calcium pyrophosphate deposition disease, Amyloidosis, Acromegaly and Mucopolysaccharidosis (resulting in carpal tunnel syndromes in children).

Others potential causes of carpal tunnel syndrome include extrinsic compression because of various anatomic constraints:

– Accessory muscles or tendons: accessory palmaris longus, accessory palmaris profundus, accessory flexor digitorum muscle or accessory lumbricals.
– Wrist synovitis or flexor tenosynovitis.
– Tumors and pseudo-tumors: Ganglion cyst of the wrist, lipoma, hamartoma, hemangioma and others mass lesions originating from the surrounding tissues.

Finally, carpal tunnel syndrome can be intrinsically caused by neurogenic neoplasm (including neurinoma and neurofibroma) [1,25,26].

Literature also emphasized the importance of dynamic factors. The most significant of these are environmental or professional conditions (prolonged postures in extremes of wrist flexion or extension, repetitive movements and exposure to impacts and vibrations). In fact, carpal tunnel syndrome remains more often an idiopathic syndrome [25,26].

### 6.2. Anatomy

The carpal tunnel is an osteofibrous canal of 6 cm in length situated in the volar wrist.

The boundaries are the carpal bones posteriorly, the tubercle of scaphoid and trapezium laterally and the pisiform and hook of the hamate medially. The anterior roof of the tunnel is the fibrous but rigid transverse carpal ligament. Also named flexor retinaculum, it is divided into two layers: one is superficial and formed by the palmaris brevis tendon, the other is deeper and made up of transversal fibers [28].

The carpal tunnel contains nine tendons (and normally no belly muscle): the flexor pollicis longus, the four flexor digitorum superficialis and the four flexor digitorum profundus tendons. The median nerve reaches the carpal tunnel and travels through it in a superficial position just below the flexor retinaculum, above the flexor tendons of the index and middle finger.

In the distal forearm (more precisely 5 cm proximal to distal transverse flexion crease of the wrist), the median nerve gives rise to the palmar cutaneous branch, which provides sensory innervation to the skin on the proximal side of the palm.

**Figure 4:**
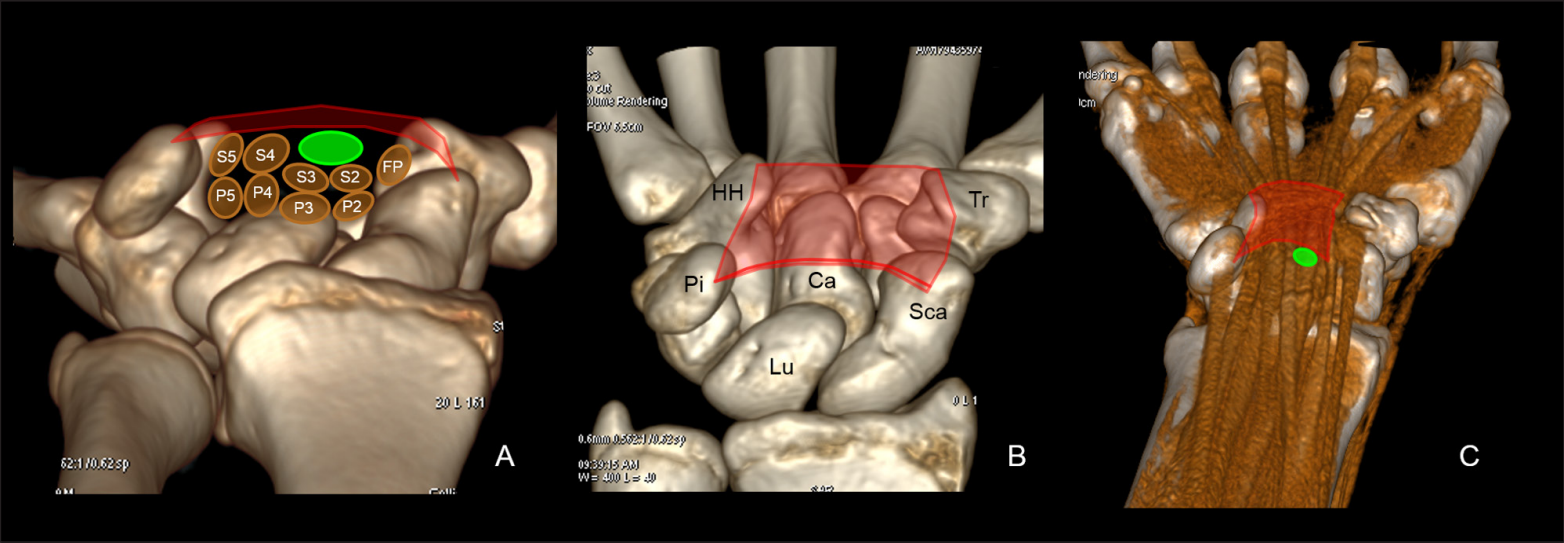
Normal anatomy of the carpal tunnel **(A, B, C)** Volume-rendered 3D computed tomography. Lu = Lunate, Ca = capitate, Sca = scaphoid, Pi = pisiform, Tr = Trapezium, HH = Hamate Hook, FP = flexor pollicis longus muscle, P = flexor digitorum profundus, F = flexor digitorum superficialis (1, 2, 3, 4) muscle. The osteofibrous canal is situated in the volar wrist and is closed anteriorly by the flexor retinaculum (red) extending from the scaphoid (Sca) and pisiform (Pi) to the trapezium (Tr) and the hook of the hamate (HH). The carpal tunnel contains nine tendons: the flexor pollicis longus (FP), the four flexor digitorum superficialis (S) and the four flexor digitorum profundus tendons (P). The median nerve (green) reaches the carpal tunnel and travels through it in a superficial position just below the flexor retinaculum and above the flexor tendons of the index and middle fingers.

Distal to the carpal tunnel, the median nerve subdivides into five branches: the recurrent motor branch to muscles of the thenar compartment and four digital sensory branches [29] (Figure 4).

**Figure 5:**
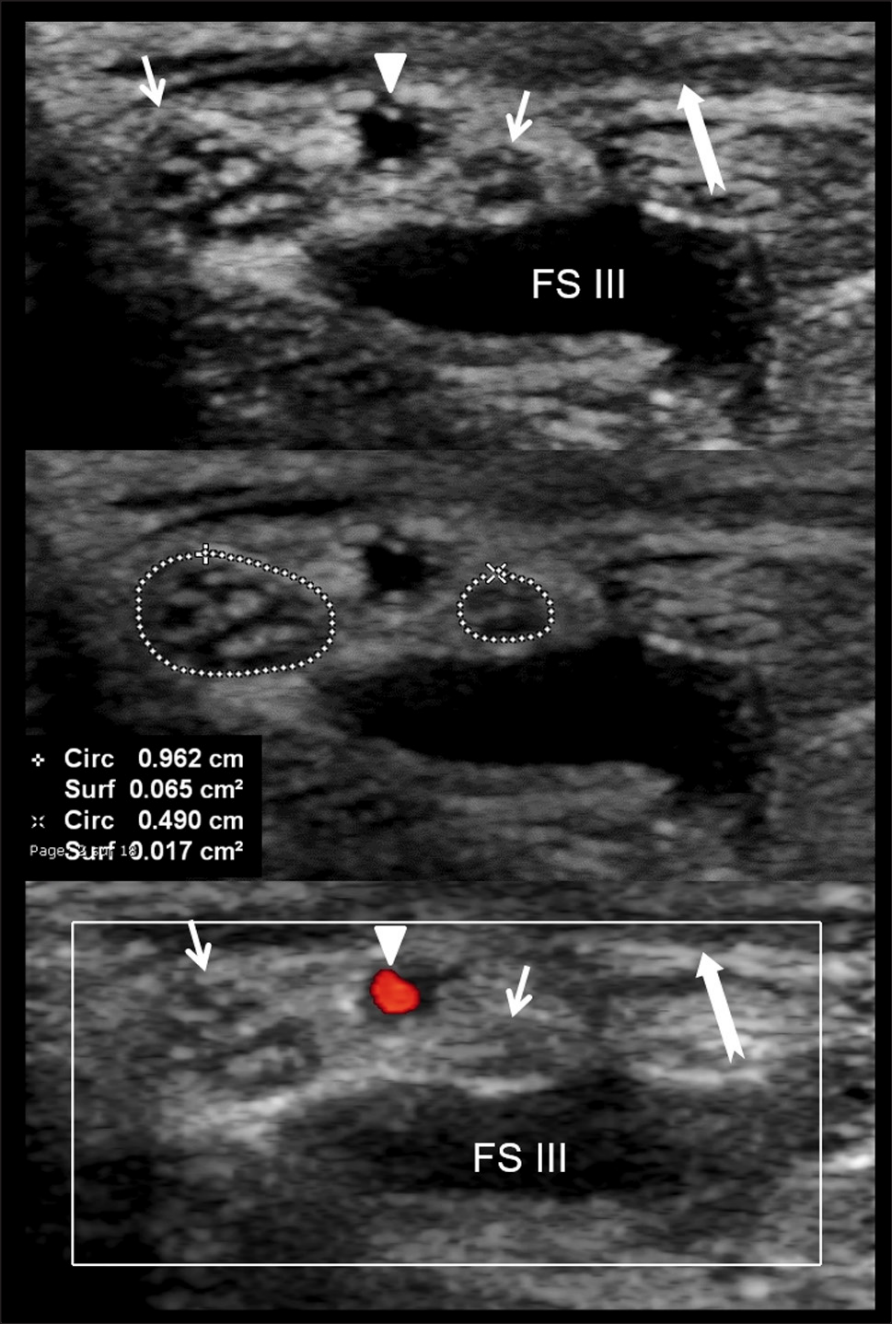
Bifid median nerve on US examination. The median nerve is divided into two bundles (small arrows) and accompanied by an accessory artery (the persistent median artery, arrowhead) which lies between the two nerve bundles. The area was 8.2 mm^2^, obtained by summing the cross-sectional area for the lateral and medial branches of the bifid median nerve. The nerve runs between the flexor retinaculum (arrow) and the flexor digitorum superficialis tendon of the middle finger (FSIII) which was hypoechogenic because of anisotropy.

The median nerve may divide into two bundles and appear as a bifid median nerve, a common variation of nerve anatomy in the carpal tunnel. A bifid median nerve may be accompanied by an accessory artery, the persistent median artery, which lies in between the two nerve bundles. Prevalence of bifid median nerve ranges from 2.8% to 18%. A bifid median nerve may be predisposed to compression in the carpal tunnel because of its relatively higher cross-sectional area compared with a nonbifid median nerve. Bifid nerves represent 0.8% to 18% of carpal tunnel syndromes [28,30,31] (Figure 5).

### 6.3. Pathophysiology

The pathophysiology involves a combination of mechanical compression and repetitive microtraumatic stretching. A normal nerve presents relatively unfettered motion during flexion and extension of the wrist and fingers, moving in the tunnel without mechanical stress [32]. Persistent nerval ischemia may induce a miniature “closed compartment syndrome” by increasing the permeability of peripheral microvessels, contributing to the development of an interstitial edema.

### 6.4. Clinical Presentation

Clinically, patients complain of paresthesia or numbness in the fingers which is worse at night and exacerbated by repetitive flexion and extension of the wrist, strenuous gripping or exposure to vibration. Nocturnal acroparesthesia is the most sensitive clinical sign (96%) [26]. Two key physical signs are associated with carpal tunnel syndrome: Tinel’s sign (abrupt tap on the volar aspect of the wrist over the carpal tunnel causing a wave of paresthesia) and Phalen’s maneuver (extreme flexion of the wrist to test for dysesthesia). Tinel’s sign is more sensitive but Phalen’s maneuver is more specific.

### 6.5. Diagnosis And Additional Exams

#### 6.5.1. Electroneuromyography

In the majority of cases, imaging is useless for diagnosis which is based on clinical grounds. However conduction studies may be used to confirm focal damage to the median nerve and to quantify the severity with an emphasis on the distal motor latency and the sensory conduction velocity [33].

Imaging has demonstrated clinical benefit in patients with uncertain diagnosis, atypical presentation, and where there is suspicion of a secondary cause or a post-surgical relapse.

#### 6.5.2. Computed Tomography And Conventional X-Ray

**Figure 6:**
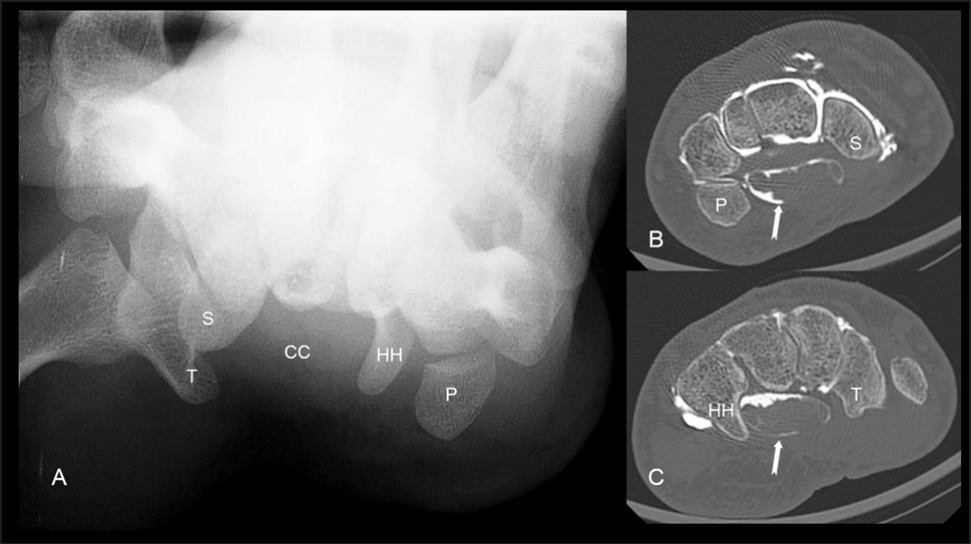
Plain radiography (**A**, carpal tunnel view in hyperextension) and axial CT views at the level of scaphoidvv/pisiform **B)** and at the level of trapezium/capitate **C)**. Sca = scaphoid, Pi = Pisiform, Tra = trapezium, Ham = hamate, CC = carpal tunnel. See the opacification of the flexor tendon sheath on the CT arthrogaphy (after injection in the mediocarpal joint).

Plain radiography (i.e. carpal tunnel view in hyperextension) might be useful in cases associated with osteophytic stenosis, fracture and soft tissue calcification. So CT scanning may provide a better alternative than plain radiography to clearly visualize the bony part of the carpal tunnel in patients with a limited range of motion (Figure 6).

#### 6.5.3. Ultrasonography

A study confirmed the usefulness of quantitative US assessment in the diagnosis of carpal tunnel syndrome in patients with negative electrodiagnostic test findings, especially in the early stage of the disease. In this study, the diagnosis was confirmed by using US examination in 31% of the cases with normal electrodiagnostic test findings [34]. The architectural alterations of the nerve studied by US precede conduction abnormalities in the early stages of nerve damage.

**Figure 7:**
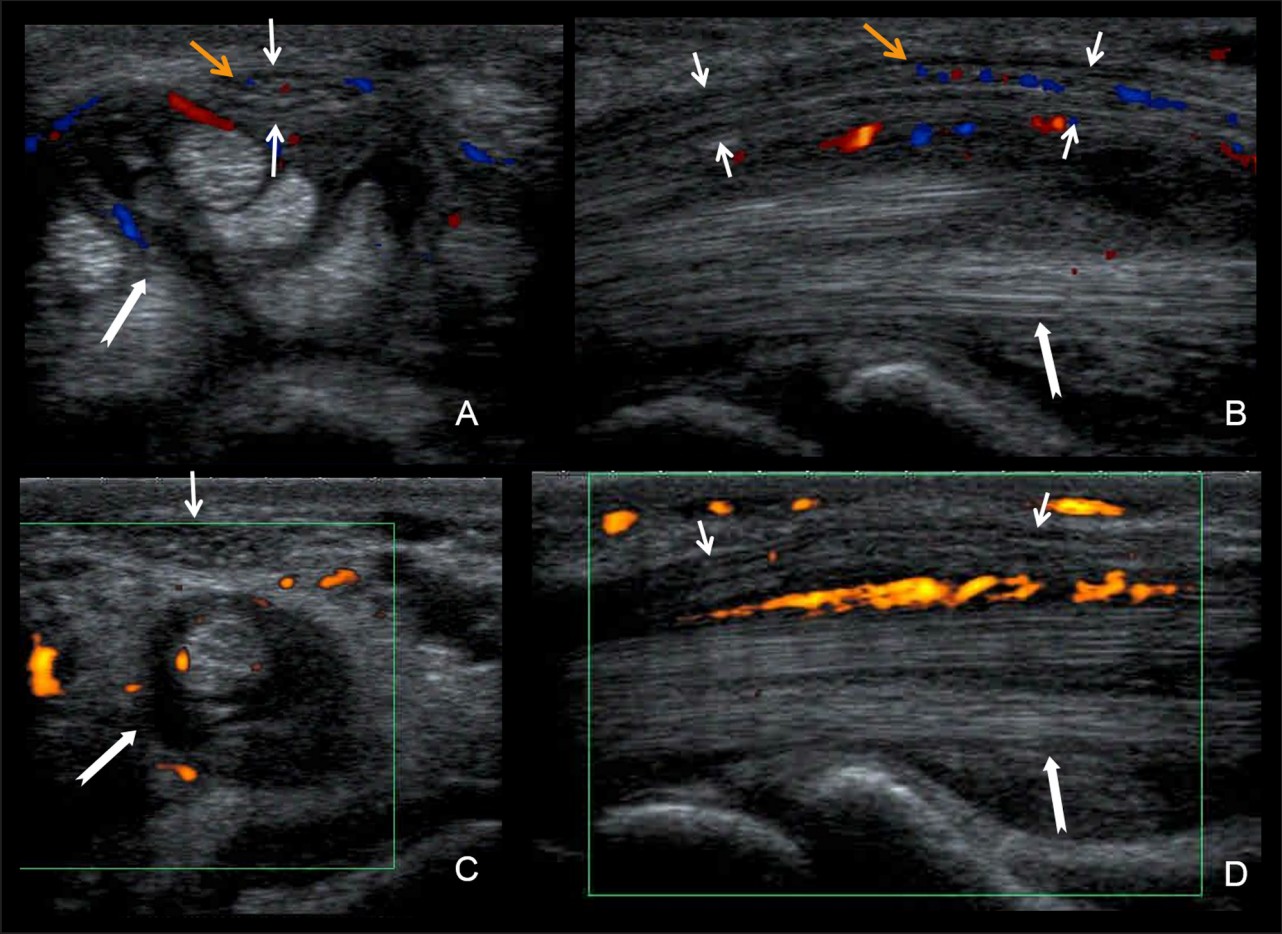
Flexor tenosynovitis. Axial **(A, C)** and sagittal **(B, D)** views on US Doppler examination. See the effusion and the hyperemia (white arrows) in the tendon sheath and the intraneural hyperemia (orange arrows) in the median nerve.

Some authors recommend to use US examination for early diagnosis and to perform subsequently further electromyography in patients with negative US. However, most experts can agree that the combination of nerve conduction studies (currently deemed the gold standard for diagnosis) and subjective symptoms allow for the most accurate way of diagnosing. If there is discordance between clinical and electrodiagnostic features or if the diagnosis remains uncertain, additional US examination can be performed [29]. The median nerve should be studied in both the short and long axis and then following up or down the extremity in the short axis (Figure 7).

**Figure 8:**
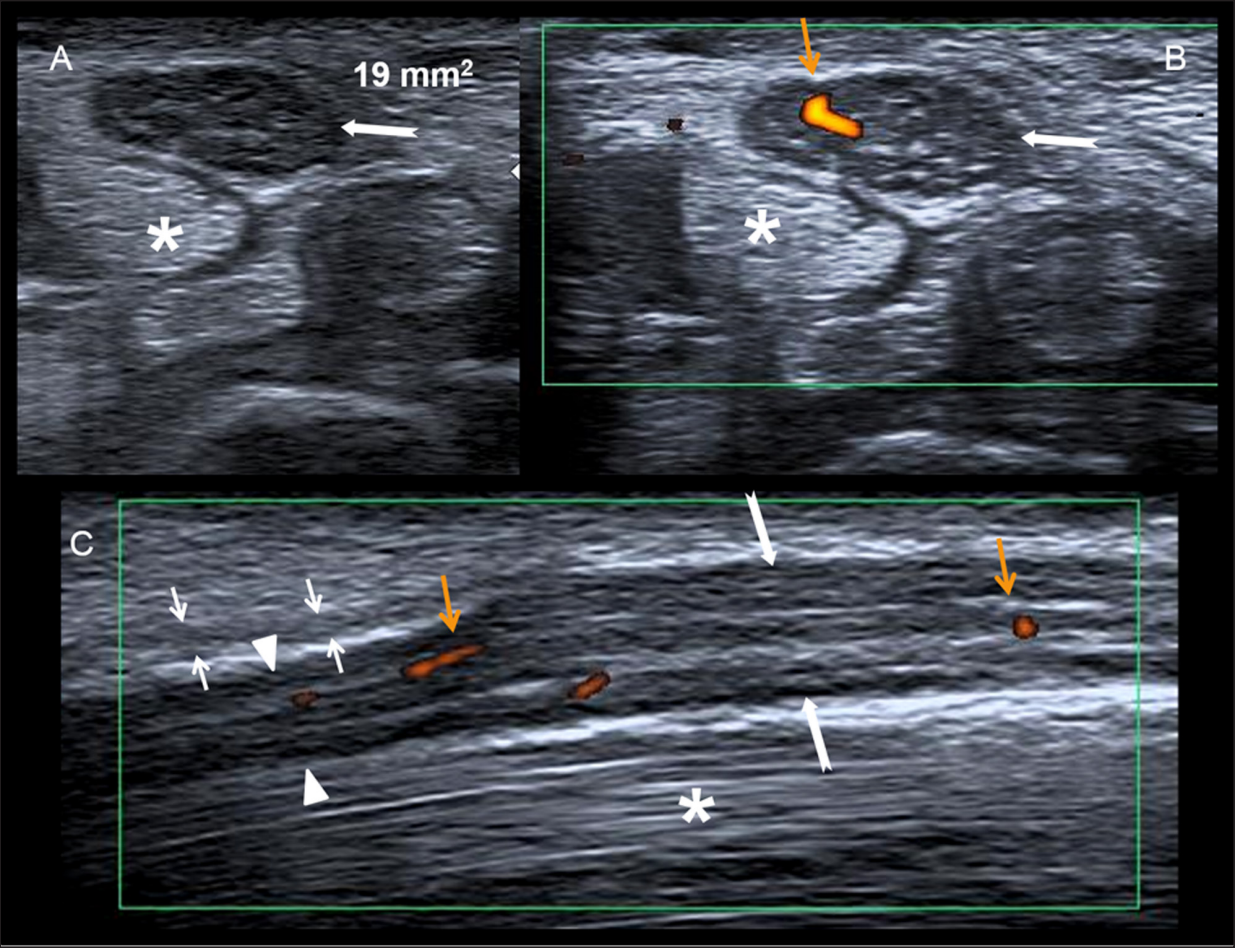
US examination of a median nerve neuropahy in a patient with carpal tunnel syndrome. Axial **(A, B)** and sagittal **C)** views. Enlargement of the median nerve (white arrows) proximal to the flexor retinaculum (small arrows) with an increased cross-sectional area (19 mm^2^). Flattening of the median nerve under the retinaculum (white arrowheads) with intraneural hyperemia (orange arrows). The flexor tendons (*) were normal.

The key diagnostic signs for carpal tunnel syndrome on ultrasound include:

– Disparity of caliber with distal flattening and proximal enlargement of the median nerve (also known as the “Notch sign”). Hourglass-like constrictions of the nerve under flexor retinaculum are described. Rarely the nerve enlargement is located distally [29,35] with a limited range of nerve motion in the sagittal plane [36] (Figure 8).
– An increased cross-sectional area of the median nerve at the level of the pisiform and scaphoid. Knowing that the normal median nerve has a cross-sectional ranging from 6.1 to 10.4 mm², a nerve size of 12 mm² has 99% sensitivity for diagnosing carpal tunnel syndrome, whereas a size of 8.5–12 mm² has 87% sensitivity [32,37,38]. In patients with bifid median nerves the optimal threshold is 12 mm^2^, obtained by summing the cross-sectional area for the lateral and medial branches of the bifid median nerve [29]. The cross-sectional area increases with the severity of disease. There is a linear relationship between nerve enlargement and reduction of conduction velocity. Normalization of the cross-sectional area appears within 2–18 months after surgical release [29,39].
– An additional cross-sectional measurement at the distal forearm with calculation of the difference improves the diagnostic accuracy of US. The difference in cross-sectional area between the nerve in the carpal tunnel (scaphoid-pisiform) and proximally at the level of the pronator quadratus muscle has better sensitivity (99%) and specificity (100%) than measurements obtained only at the level of the carpal tunnel, with an optimal cut off value of 2 mm². In patients with bifid median nerve, the optimal threshold for the difference is 4 mm² (specificity > 90%) [30,31,40].
– Intraneural hyperemia due to a compression of the median nerve with congestion of epineural and endoneural veins, nerve oedema and impairments of blood supply [41].

Other ultrasound findings are described in carpal tunnel syndrome but appear to have low accuracy:

– A hypoechogenic median nerve with loss of its characteristic fascicular appearance.
– Changes in the flexor retinaculum consisting in thickening and bowing (>4 mm beyond the line joining the hamate hook to the trapezium).
– Calculation of the flattening ratio (i.e. long axis/short axis measurements ratio on an axial view). A value exceeding 4 is associated with carpal tunnel syndrome [28].

Unfortunately, US examination is normal in 20% of the patients at the early stage of the disease. Measurement of the maximal cross-sectional area obtained only at the level of the carpal tunnel may be sufficient if the median nerve shows a significant enlargement. If the nerve enlargement is moderate, a combination of numerous ultrasound findings improves the diagnostic accuracy. If a clinical recurrence is suspected after surgery, US can detect an incomplete release of the flexor retinaculum [28] or even a perineural fibrosis (irregular hypoechogenic rim surrounding the median nerve). Dynamic evaluation may also show a limited range of nerve motion because of adhesions between the nerve and the adjacent structures [35].

#### 6.5.4. Magnetic resonance imaging

Diagnostic criteria for carpal tunnel syndrome on MRI are similar to those on ultrasound: namely, bowing of the flexor retinaculum, enlargement of the proximal median nerve and flattening of the distal nerve. Increased signal of the nerve on T2-weighted sequences is also described, due to nerve edema and focal demyelination. However in clinical practice, the detection of signal changes indicating an entrapped median nerve depends mainly on visual interpretations by radiologists, making it subject to observer variation [42]. A decreased signal on T2-weighted sequences due to fibrosis can be lately visible in patients with longstanding disease. Some cases will exhibit enhancement of the nerve after administration of IV gadolinium, due to edema. In contrast, fibrosis and ischemia could explain the non-enhancement [43].

**Figure 9:**
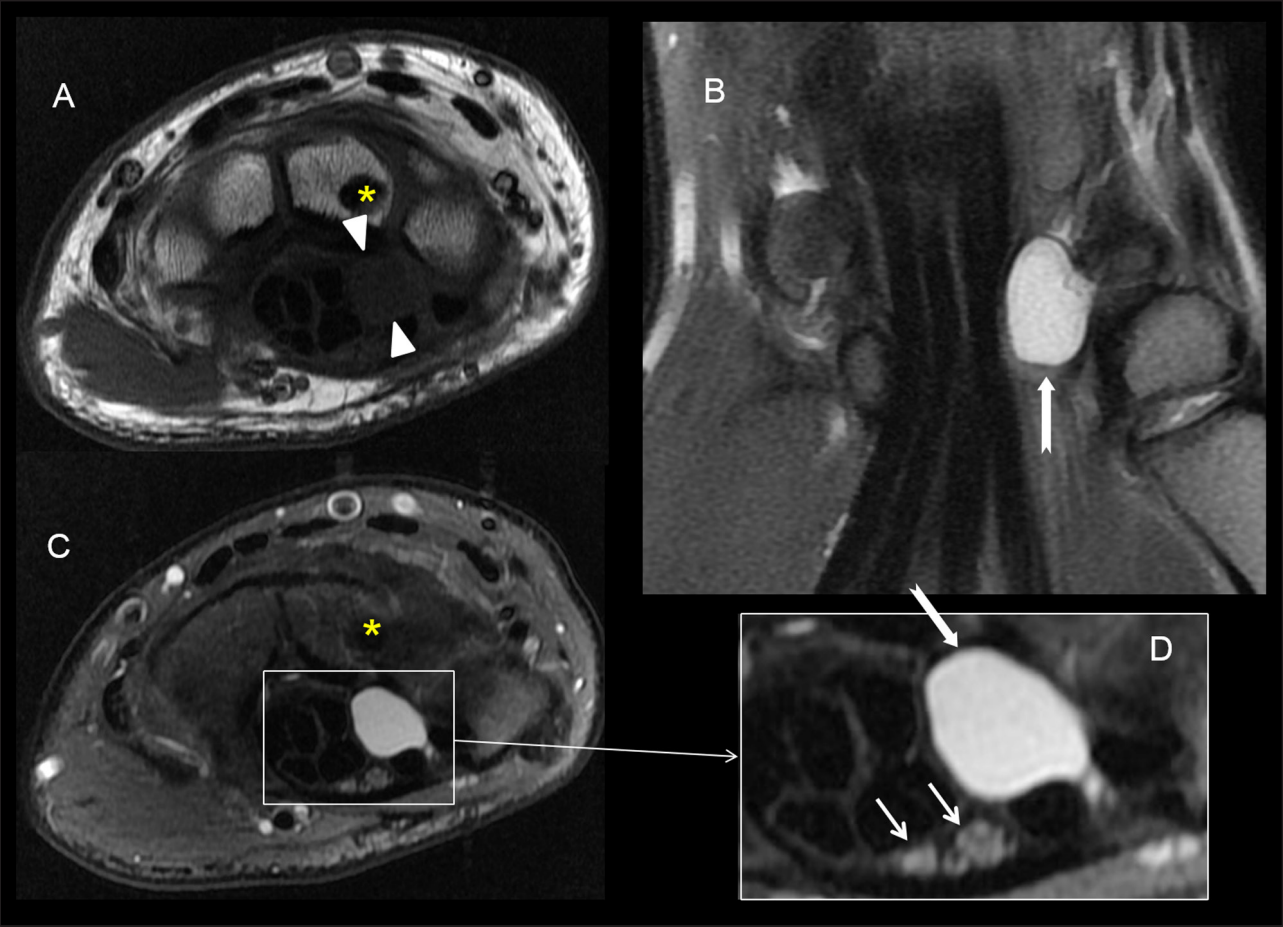
Median nerve compression due to a ganglion cyst originating from the joint. T1-weighted axial image **A)** and T2-weighted axial **(C, D)**, coronal **B)** and sagittal **E)** images with Fat Sat. T1-weighted sagittal image with FS after injection of gadolinium (F). MRI showed a space-occupying mass with fluid-like signal (low signal on T1 (white arrowheads) and high signal on T2-weighted images (white arrows) located at the deep and lateral part of the carpal tunnel. It corresponded to a ganglion cyst originating from the ventral scapholunate interval. The cyst caused an extrinsic compression of the bifid median nerve (small white arrows) which showed an increased signal on T2-weighted images (yellow arrowheads) and an enhancement after the IV administration of gadolinium (yellow arrows). The ganglion cyst presented a discrete and peripheral enhancement (black arrowheads). Note the presence of an incidental enostosis (yellow asterisk).

**Figure 10:**
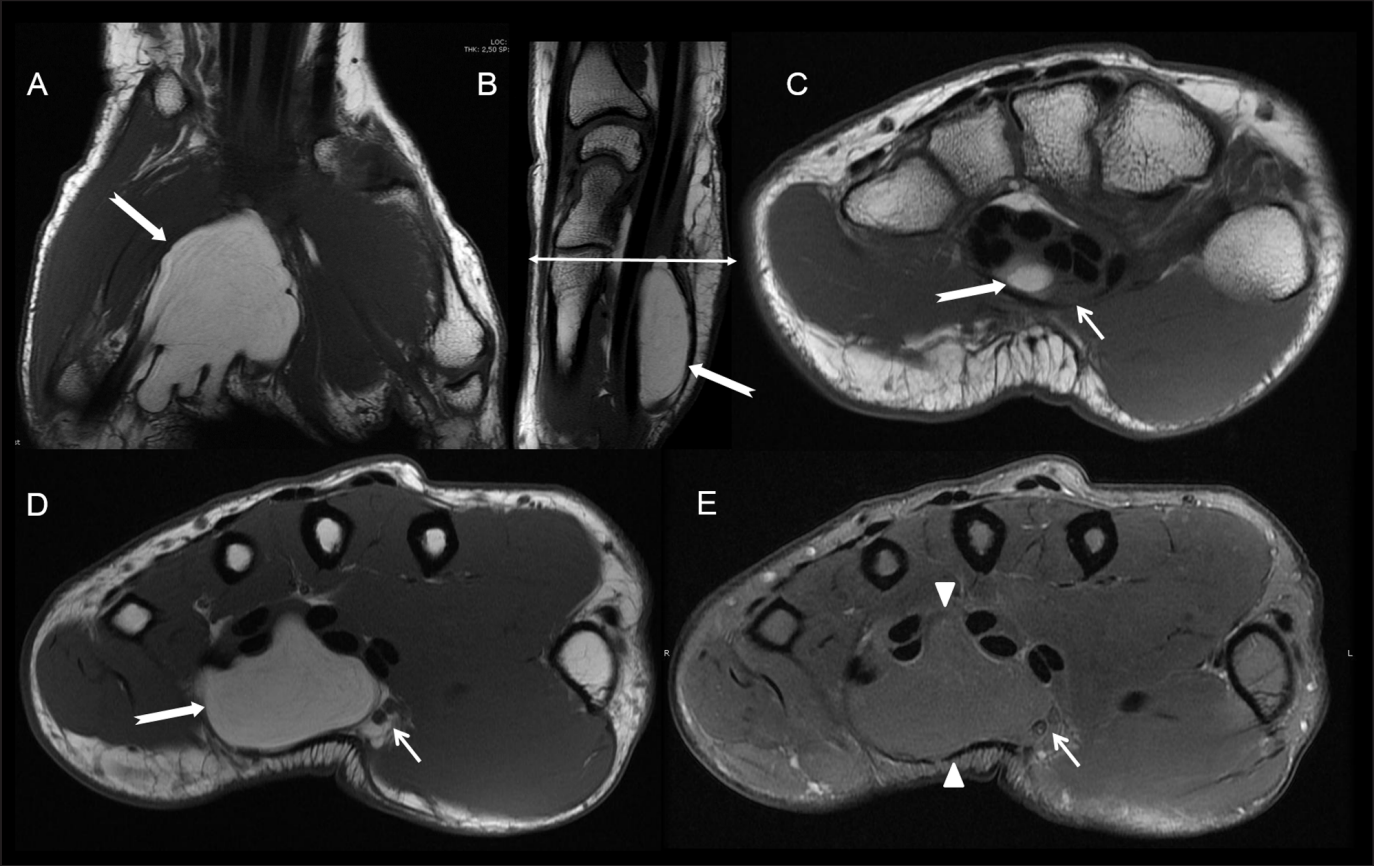
Carpal tunnel syndrome caused by a lipoma. MRI with T1-weighted coronal **A)**, sagittal **B)** and axial **(C, D)** images and with T2-weighted FS axial **E)** image. MRI showed a space-occupying mass in the distal carpal tunnel with fat-like signal (increased signal in T1-weighted images (white arrows) with saturation on T2 FS image (arrowheads)) causing a compression of the median nerve and its branches (small white arrows).

As on US examination, the role of MRI is also to detect extrinsic or intrinsic causes with a better diagnostic accuracy for mass lesions than US (especially for tumoral or pseudotumoral nerve lesions) (Figures 9 and 10).

3T MR neurography by using diffusion-tensor imaging (DTI) allows assessment of the nerve continuity and neural micro-architecture. Fractional anisotropy (FA) of the median nerve is both a sensitive and specific predictor of carpal tunnel syndrome using DTI. A difference superior to 0.058 between FA measured at the level of the radiounlar joint and FA of the median nerve in distal carpal tunnel is an accurate diagnostic criteria for median nerve damage [44,45,46].

Post-operative MRI is useful for assessing the degree of nerve damage after treatment, in association with electromyography. In particular, the aim of post-operative MRI is both to detect a residual mass lesion and to analyze the carpal tunnel release by studying the scar tissue and fibrosis on T1-weighted sequences after injection of gadolinium [47].

## 7. Conclusion

Carpal tunnel syndrome is the most common entrapment neuropathy and usually has a typical clinical presentation. Nerve conduction studies may be used in equivocal or incomplete presentations to confirm focal damage to the median nerve and to quantify the severity of the neuropathy. However it must be remembered that electromyography may be completely normal in patients with a clinical syndrome, especially in the early stage. This emphasizes the importance of imaging (and in particular US examination) in providing strong arguments for diagnosis: namely, disparity of the nerve under the flexor retinaculum, intraneural hyperemia, increased cross-sectional area of the median nerve (>12 mm²), and difference in cross-sectional area (Δ > 2 mm²) between the nerve at the wrist and at the forearm. Moreover, US is also an accurate and simple way to detect the main causes of the secondary forms (such as anatomical variations and space-occupying lesions) which may guide management of the patient.
